# A combined laparoscopic and vaginal approach with a novel method to reconstructing the uterine cervix in Herlyn-Werner-Wunderlich syndrome with cervical obstruction: A case report

**DOI:** 10.1016/j.ijscr.2024.110029

**Published:** 2024-07-14

**Authors:** Mami Shibahara, Kaori Hoshino, Hiroshi Harada, Taeko Ueda, Tomoko Kurita, Kiyoshi Yoshino

**Affiliations:** Department of Obstetrics and Gynecology, University of Occupational and Environmental Health, Kitakyushu, Japan

**Keywords:** Herlyn-Werner-Wunderlich syndrome, Uterine anomalies, Gynecologic surgery, Cervical atresia, Obstructed hemivagina and ipsilateral renal anomaly syndrome, Case report

## Abstract

**Introduction:**

Herlyn-Werner-Wunderlich syndrome (HWWS) is characterized by uterine didelphys, unilateral cervical obstruction, and ipsilateral renal defects. Owing to its rarity, no standard surgical approach exists.

**Presentation of case:**

An 11-year-old girl with severe dysmenorrhea had a duplicated uterus, a right cervical hemorrhagic cyst, and right ipsilateral kidney agenesis, indicative of HWWS. As transvaginal surgery was challenging, we turned to laparoscopic surgery for abdominal cavity inspection and surgical assistance. A longitudinal incision was made on the right uterus, followed by inserting a catheter tube fixed to an intrauterine device (IUD) into the right cervical canal from the anterior wall of the right uterine horn. Subsequently, the right external cervical os was inverted to prevent restenosis. Postoperatively, the hemorrhagic cyst at the right cervix disappeared. The patient had no symptom recurrence 24 months after the surgery.

**Discussion:**

The preoperative diagnosis for female genital malformations is complicated, and transvaginal manipulation is often difficult in adolescent girls. Laparoscopy is a valuable tool for evaluating female genital malformations, allowing for a thorough diagnosis and safe surgical treatment. In cases of female genital malformation with cervical obstruction, as in this case, reconstruction of the uterine cervix is important to prevent restenosis after surgery.

**Conclusion:**

In female genital malformations, laparoscopy provides a comprehensive evaluation of the malformation, assisting in a precise diagnosis and safe surgical treatment. Insertion of the catheter tube with IUD into the uterus and reconstruction of the cervix contribute to preventing restenosis.

## Highlights

• A typical surgical procedure for Herlyn-Werner-Wunderlich syndrome with cervical obstruction has not yet been established.
• We devised a new method by a combined laparoscopic and vaginal approach using a catheter tube fixed to an intrauterine device.
• A thorough evaluation of the malformation and visualization of the abdominal cavity is necessary for safe surgical treatment.
• Particular attention should be given to patients with cervical obstructions to prevent restenosis after surgery.

## 1 Introduction

Female genital malformations have numerous presentations. Consequently, standard surgical approaches have yet to be established. Among asymmetric uterine malformations with unilateral renal aplasia, Herlyn-Werner-Wunderlich syndrome (HWWS) and obstructed hemivagina and ipsilateral renal anomaly (OHVIRA) syndrome have been reported. Although the malformations of these diseases are similar, it is important to distinguish them owing to the different approaches to their treatment.

In 1971, Herlyn-Werner syndrome, a malformation featuring a duplicated uterus, a unilateral Gartner cyst with traffic in the uterine lumen, and ipsilateral renal aplasia, was reported [1]. In 1976, Wunderlich syndrome was first described as a disease characterized by a duplicated uterus, unilateral cervical obstruction, and an ipsilateral renal defect [2]. Since then, disorders similar to these two syndromes have often been collectively reported as HWWS. The primary symptoms of HWWS include dysmenorrhea and abdominal pain after menarche, especially in cases with complete obstructions [3]. More recently, in 2007, OHVIRA syndrome, a condition identified by a duplicated uterus, an obstructed hemivagina, and an ipsilateral renal aplasia, was reported [4]. Abdominal pain is the main symptom of OHVIRA, similar to HWWS [4]. Despite their similarities, the surgical approaches and outcomes for HWWS and OHVIRA syndromes differ. An obstructed hemivagina (i.e., OHVIRA syndrome in the narrow sense) can be treated with wide resection of the vaginal septum and drainage. However, in HWWS cases where the cervix is obstructed, a wide resection of the cervix is not feasible, and a more advanced surgical approach, such as cervical reconstruction, is required. Moreover, the method used to reconstruct the cervix must also be taken into account to prevent restenosis. In addition, symptom onset due to obstructive genital malformations is often observed in young patients soon after menarche, and a narrow vagina complicates the successful completion of surgery by transvaginal manipulation. When performing surgery proves challenging through transvaginal manipulation alone, the assistive use of a laparoscope can facilitate surgical success and safety.

Here, we detail the case of a patient who underwent laparoscopically assisted cervical reconstruction to address HWWS with cervical obstruction. In addition, we outline a proposed method for a safe surgical approach and characteristic devices for the prevention of cervical restenosis. This case is written in line with the SCARE 2023 guideline [5].

## 2 Case report

**Fig. 1:**
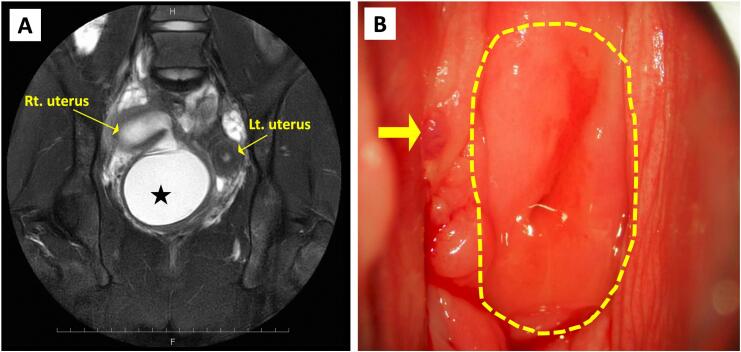
(**A**) Magnetic resonance imaging (T2-weighted imaging, coronal plane) of a duplex uterus and hemorrhagic cyst (black star) below the right uterus. (**B**) Vaginal examination of the left cervix (broken yellow line) and the trace of drainage on the right side (yellow arrow). (For interpretation of the references to colour in this figure legend, the reader is referred to the web version of this article.)

An 11-year-old girl presented to a primary hospital with severe dysmenorrhea. She had menarche five months before the visit, and since then, she had been experiencing severe cyclical abdominal pain. Transabdominal ultrasound and magnetic resonance imaging revealed a duplicated uterus, a hemorrhagic cyst below the right uterus, and right kidney agenesis (Fig. 1A). However, imaging was unable to determine whether the patient had an obstructed hemivagina or cervical atresia of the right side. The patient underwent transvaginal drainage twice under general anesthesia at the primary hospital, resulting in repeated infections and restenosis. Subsequently, the patient was admitted to our department for advanced surgery.

**Fig. 2:**
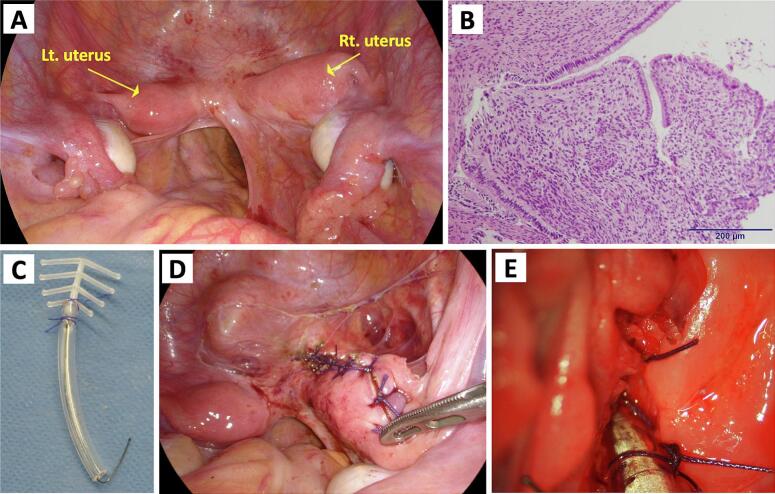
(**A**) Intraoperative laparoscopic image. (**B**) Histopathological findings at the site of stenosis reveal cervical columnar epithelium (hematoxylin and eosin). (**C**) An intrauterine device (FD-1) fixed to a catheter tube with absorbable thread. (**D**) The sutured right uterus after insertion of the FD-1 intrauterine device with catheter tube. (**E**) The reconstructed right cervix.

During our surgery, the left cervix was visible on examination; however, the right cervix was not discernible, and only a trace of drainage remained (Fig. 1B). We attempted transvaginal drainage guided by transabdominal ultrasonography, but this proved difficult because of fibrotic scar tissue that had formed from previous treatments. Given the risk of injury to other organs, a laparoscope was deployed to inspect the abdominal cavity and assist with the surgery. Laparoscopy revealed a duplicate uterus (Fig. 2A) and an endometriotic lesion in the pouch of Douglas without any adhesions. We were unsuccessful transvaginally in reaching the endometrial cavity, even under endoscopic monitoring, owing to the persistent cervical stenosis. Therefore, we opted to reach the endometrium by making an incision on the anterior wall of the right uterine horn and address the cervical stenosis from above. We performed a biopsy on the stenosis. Intraoperative pathology revealed cervical columnar epithelial tissue (Fig. 2B); thus, the patient was diagnosed with HWWS complicated by cervical obstruction. Accordingly, we decided to preserve and reconstruct the right cervix instead of removing the stenotic site.

A catheter tube was inserted into the right cervical canal from the anterior wall of the right uterine horn. The catheter tube was fixed to an FD-1 (Fuji Latex, Tokyo, Japan) intrauterine device (IUD) with absorbable thread (Fig. 2C) using the method we had previously devised and reported [6]. The catheter will be removed in the future, leaving the IUD by itself to maintain external cervical os patency. After placing the catheter tube in the cervical canal, the uterine incision was closed (Fig. 2D). Finally, the right cervical canal was everted from the vaginal side to prevent restenosis (Fig. 2E). Postoperatively, the right cervical hemorrhagic cyst disappeared. Four months postoperatively, only the catheter tube was removed, leaving the IUD in the uterus. The patient had no symptom recurrence 24 months after the surgery.

## 3 Discussion

**Fig. 3:**
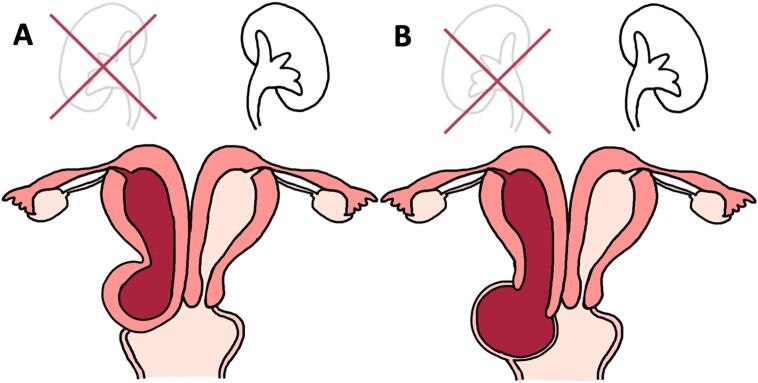
Illustration of the genital anomalies. (**A**) HWWS with cervical obstruction (this case). (**B**) Obstructed hemivagina and ipsilateral renal anomaly syndrome (in the narrow sense).

We performed laparoscopically assisted cervical reconstruction on an adolescent girl who had HWWS with cervical obstruction. The preoperative diagnosis for malformations, as seen in this particular case, is complicated. Fig. 3 shows the malformations of HWWS with cervical obstruction (Fig. 3A) and OHVIRA syndrome in the narrow sense (Fig. 3B). In the present case, imaging studies revealed a duplicate uterus, right renal aplasia, and a hemorrhagic cyst below the right uterus. Imaging results are not sufficient to distinguish HWWS with cervical atresia from OHVIRA syndrome with vaginal obstruction. However, it is imperative to differentiate between these syndromes owing to the varying surgical strategies involved, as outlined in the following paragraph. Pathological confirmation of the tissue at the stenosis site may be necessary to arrive at a definite diagnosis. OHVIRA syndrome is associated with vaginal obstruction, and the septum is composed of a stratified squamous epithelium. In contrast, in the case of HWWS with cervical atresia, the tissue from the affected site is composed of tall columnar epithelium from the cervical glands. In the present case, the tissue from the stenotic site yielded cervical columnar epithelial tissue; therefore, the patient was diagnosed with HWWS with cervical obstruction. The European Society of Human Reproduction and Embryology/European Society of Gastrointestinal Endoscopy Classification [7] categorizes this type of malformation as U3B/C3/V0 (Fig. 3A).

No uniform surgical approach has been established for congenital uterine malformations, particularly obstructive genital malformations. Treatment approaches and prognoses differ depending on the extent of malformations and the obstruction site. Wide resection of the vaginal septum is effective and sufficient in cases of vaginal obstruction. Conversely, in cases of cervical obstruction, the extensive resection of the affected cervix is unfeasible, and merely enlarging the cervical orifice can lead to restenosis. Therefore, these malformations are associated with a poor prognosis [3]. Although there are some case reports of hemihysterectomy for a patient with HWWS [8,9], the surgery is more invasive; therefore, we consider cervical reconstruction as the best treatment. However, reconstructing the cervix and keeping it intact require some ingenuity. We then devised some surgical procedures. We used a catheter tube fixed to the IUD with an absorbable thread. This enables us to remove only the catheter tube in the future while leaving the IUD in place to ensure the patency of the external cervical os [6]. This strategy effectively maintains a sufficiently wide cervical diameter to prevent restenosis. Moreover, we inverted the edge of the cervix to further prevent recurrence. We initially developed this technique based on the construction of a neocervix in radical trachelectomy [10].

Although there is no specific evidence regarding the appropriate timing for removing the catheter tube and IUD, these devices should be managed as follows. The catheter tube should not be left in place for prolonged periods, given the potential risk of intrauterine infections. Moreover, periodic exchanges of the catheter tube are not feasible on an outpatient basis for girls who have not had their first intercourse. In contrast, an IUD can be implanted for an extended period. Therefore, we removed the catheter tube a few months after surgery and left the IUD in the uterus. The thread attached to the IUD extends from the cervix to the vaginal side, which helps prevent complete restenosis of the cervix. In case of restenosis, this also facilitates locating the external cervical os. Due to incision in the affected uterus, future pregnancies in the affected uterus would be high risk. Therefore, the IUD will remain implanted to provide contraceptive effects. We plan to replace the IUD once the patient is old enough for outpatient internal examinations. The reproductive ability and pregnancy prognosis after this surgery are unknown due to a lack of precedent. We expect that vaginal delivery is feasible for pregnancies in the normal side of the uterus. However, a previous study has reported that pregnancies with uterine anomalies are more likely to be associated with miscarriage, preterm labor, abnormal fetal position, and a higher rate of cesarean section [11]. These findings indicate the necessity of individual case-specific considerations. Long-term follow-up is necessary to assess pregnancy outcome and to confirm whether cervical patency can be maintained after tube removal.

In adolescent girls, transvaginal manipulation is often difficult and carries the risk of injury to other organs. Some case reports have described the use of laparoscopy for transvaginal drainage [[12], [13], [14]]. In the present case, transvaginal manipulation was challenging, and a laparoscope was used to inspect the abdominal cavity and prevent organ injury. Further, we were unable to introduce a catheter tube via the transvaginal approach, even under endoscopic monitoring; therefore, we inserted it from the uterine corpus through an incision in the anterior wall of the affected uterus. Laparoscopy is a valuable tool for evaluating female genital malformations, allowing for a thorough diagnosis and safe surgical treatment. The applicability and outcomes of this technique should be validated in a larger patient cohort in future studies.

## 4 Conclusion

Although a typical surgical procedure for HWWS especially with cervical obstruction has not yet been established, we devised a new surgical approach based on previous methods and successfully performed a safe and effective operation. Based on this case and previous similar case reports, we consider that a thorough evaluation of the malformation is necessary and that visualization of the abdominal cavity with laparoscopy is advantageous for precise diagnosis and safe surgical treatment. Particular attention should be given to patients with cervical obstructions to prevent restenosis after surgery. Insertion of catheter tube with IUD into the uterus and reconstruction of the cervix contribute to preventing restenosis. Further studies should evaluate the technique's applicability and outcomes in a larger patient cohort.

## Patient consent

Written informed consent was obtained from the patient and the patient's parents for publication and any accompanying images. A copy of the written consent is available for review by the Editor-in-Chief of this journal on request.

## Ethical approval

Ethical approval is not needed for case reports in our institution (Ethics Committee of Medical Research, University of Occupational and Environmental Health, Japan).

## Funding

Not applicable.

## Author contribution

MS and KH drafted and revised the manuscript. TK proposed the surgical approach for this case. HH, TU, and KY made substantial contributions to revise the manuscript critically for intellectual content. All authors read and approved the final manuscript.

## Guarantor

Kaori Hoshino.

## Research registration number

Not applicable.

## Conflict of interest statement

The authors have no potential conflicts of interest to disclose.
